# Chromatin modifications and genomic contexts linked to dynamic DNA methylation patterns across human cell types

**DOI:** 10.1038/srep08410

**Published:** 2015-02-12

**Authors:** Haidan Yan, Dongwei Zhang, Hongbo Liu, Yanjun Wei, Jie Lv, Fang Wang, Chunlong Zhang, Qiong Wu, Jianzhong Su, Yan Zhang

**Affiliations:** 1 College of Bioinformatics Science and Technology, Harbin Medical University, Harbin 150081, China; 2 The Department of General Surgery, The Second Affiliated Hospital, Harbin Medical University, Harbin 150086, China; 3 School of Life Science and Technology, State Key Laboratory of Urban Water Resource and Environment, Harbin Institute of Technology, Harbin 150001, China

## Abstract

DNA methylation is related closely to sequence contexts and chromatin modifications; however, their potential differences in different genomic regions across cell types remain largely unexplored. We used publicly available genome-scale DNA methylation and histone modification profiles to study their relationships among different genomic regions in human embryonic stem cells (H1), H1-derived neuronal progenitor cultured cells (NPC), and foetal fibroblasts (IMR90) using the Random forests classifier. Histone modifications achieved high accuracy in modelling DNA methylation patterns on a genome scale in the three cell types. The inclusion of sequence features helped improve accuracy only in non-promoter regions of IMR90. Furthermore, the top six feature combinations obtained by mean decrease Gini were important indicators of different DNA methylation patterns, suggesting that H3K4me2 and H3K4me3 are important indicators that are independent of genomic regions and cell types. H3K9me3 was IMR90-specific and exhibited a genomic region-specific correlation with DNA methylation. Variations of essential chromatin modification signals may effectively discriminate changes of DNA methylation between H1 and IMR90. Genes with different co-variations of epigenetic marks exhibited genomic region-specific biological relevance. This study provides an integrated strategy to identify systematically essential epigenetic and genetic elements of genomic region-specific and cell type-specific DNA methylation patterns.

In mammals, DNA methylation is a well-known epigenetic modification that plays major roles in gene transcription regulation^1^. Abnormal DNA methylation is involved in multiple human cancers^2^,^3^,^4^,^5^ that almost always display an overall DNA methylation loss of gene bodies but exhibit specific hypermethylation at gene promoter CpG islands^6^,^7^. During the process of human stem cell differentiation and development, DNA methylation is a critical mark that defines cellular identity and the development state^8^,^9^,^10^,^11^.

DNA methylation almost always takes place in the cytosine residues of CpG dinucleotide symmetrically on both strands of the human genome^12^. Genome-scale DNA methylation profiles show that most CpG dinucleotides are methylated throughout the genome except in CpG-rich CpG islands^13^,^14^. Genomic sequence contexts contain important genetic information that can distinguish DNA methylation patterns^15^,^16^,^17^, but it is difficult to comprehensively explain extensive DNA methylation changes during cellular differentiation, tumourigenesis, or aging based only on sequence characteristics. For example, the promoter of the pluripotency gene *Oct4* (also known as *Pou5f1*) is hypomethylated in embryonic stem cells but becomes hypermethylated in foetal fibroblast (IMR90) cells^18^. Most recent studies have reported that histone modifications were closely connected to DNA methylation through biochemical interactions^14^,^19^,^20^,^21^,^22^. In mammals, the histone methyltransferase Suv39h is required to establish histone H3 lysine 9 (H3K9) methylation, which is required to direct DNA methylation of pericentric satellite repeats^23^. Similarly, the histone methyltransferase G9a is a master regulator required for H3K9 methylation and DNA methylation^24^,^25^. Methyl-CpG-binding proteins that interact with methylated DNA have been linked with histone deacetylases^26^,^27^. Further, genes that are de novo methylated in cancer cells are enriched with trimethylated histone H3 lysine 27 (H3K27me3)^28^. Therefore, it is of interest to investigate the DNA methylation dynamics among different cell types by integrating sequence features and histone modifications.

Computational and experimental approaches have been used to investigate the correlation between DNA methylation and histone modifications and/or sequence features. A strong negative correlation was reported between DNA methylation and H3K4me3 at promoters and CpG islands^29^,^30^,^31^. Moreover, genes with unmethylated CpG-rich promoters enriched with H3K4me3 were found to play important roles in regulating embryonic development, and some of these genes were house-keeping genes^32^. Conversely, methylated CpG-poor promoters devoid of H3K4me3 were reported to be enriched preferentially in tissue-specific genes^29^,^30^. These computational analyses found that DNA methylation was linked closely with histone modifications and that their interactions may have important biological functions. However, these analyses focused only on CpG islands and promoters. Experimental approaches including high-throughput sequencing of bisulfite-treated chromatin immunoprecipitated DNA (BisChIP-seq)^33^,^34^ and single-molecular analysis^35^, can directly detect genomic regions where DNA methylation and certain histone modifications are mutually exclusive or co-occur. For example, Statham et al.^34^ found that the relationship between H3K27me3 and DNA methylation was genomic-region dependent (i.e., in normal epithelial cell, H3K27me3 occurred in both unmethylated and methylated regions within transcription start sites (TSSs) and CpG islands, whereas, in the rest of the genome, H3K27me3 associated only with methylated regions) and was different between normal somatic epithelial cell and prostate cancer cell. In contrast, Brinkman et al.^33^ found that H3K27me3 and DNA methylation were mutually exclusive in high CpG density genomic regions such as CpG islands but co-occur throughout most of the genome in both human colon cancer HCT 116 cells and mouse embryonic stem cells. The different conclusions may be caused by different cell types, suggesting DNA methylation of specific cell types may have specific associations with H3K27me3 in particular genomic regions. These experimental studies investigated only the relationships between DNA methylation and repressive histone modifications such as H3K27me3 and H3K9me3. Therefore, active marks closely involved with gene expression were not explored.

In this paper, high-throughput DNA methylation profiles based on the bisulfite-sequencing (BS-seq) technique and 16 histone modification profiles based on the chromatin immunoprecipitation followed by sequencing (ChIP-seq) technique in human embryonic stem cells (H1), H1-derived neuronal progenitor cultured cells (NPC), and foetal fibroblasts (IMR90) were collected. We identified whole genome-wide DNA methylation patterns and divided them into four different genomic regions in the three cell types. Random forests was applied to comprehensively analyze the relationships between DNA methylation patterns and histone modifications and sequence features in different genomic regions and in the three cell types.

## Results

### Genomic context biases and cell line-specific chromatin modification signals of DNA methylation patterns

**Figure 1:**
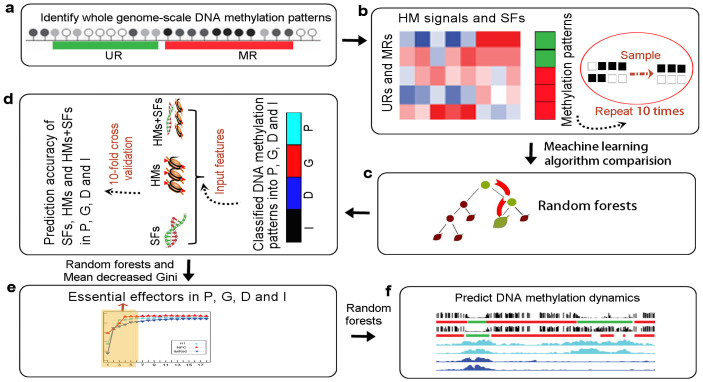
Framework used to identify genomic-region specific and cell type-specific relationships between DNA methylation and histone modifications/sequence features. (a) Identification of whole genome-scale unmethylated regions (URs) and methylated regions (MRs) from high-throughput DNA methylation profiles. (b) Calculated sequence features (SFs) and histone modification (HM) signals of DNA methylation patterns. To avoid data bias, we sampled the same numbers of unmethylated and methylated regions by 10 times repetition for the machine learning algorithms. (c) Comparison of the performances of five frequently-used classification algorithms (Random forests, RBF-SVM, decision tree J48, naive Bayes, and logistic regression) for DNA methylation prediction. 10-fold cross validation was applied to each of the 10 sampled datasets to obtain reliable model accuracy. Random forests achieved the best performance and was applied to the following analysis. (d) DNA methylation patterns were grouped into four genomic regions: promoter (P), gene body (G), downstream (D), and intergenic (I) regions. The prediction accuracies of sequence features, histone modifications, and the combination of histone modifications and sequence features (HMs + SFs) were estimated in the promoter, gene body, downstream, and intergenic regions. (e) Identification of essential effectors of DNA methylation in the promoter, gene body, downstream, and intergenic regions by comparing the prediction accuracies of the top *n* most important feature combinations. The relative importance of each feature was evaluated by mean decrease Gini and ordered from most important to least important. (f) Essential histone modifications used to predict DMR patterns (from unmethylated to methylated patterns and from methylated to unmethylated patterns). DMR patterns also were classified into the four genomic regions between paired cell types.

The characteristics of DNA methylation have been shown to be linked closely with histone modifications^14^,^36^ and the underlying genomic contexts^16^. Our study provides an integrated analysis framework to explore these relationships in different genomic regions and in various cell types (Figure 1), which may improve the understanding of their interactions.

**Figure 2:**
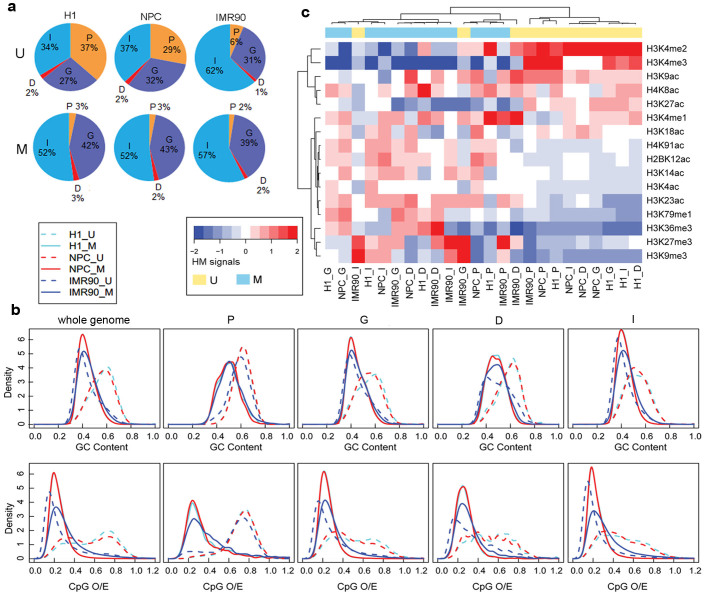
Distribution of methylation patterns, sequence features, and histone modifications in three cell lines. (a) Identified unmethylated and methylated regions were annotated in the promoters (P), gene body (G), downstream (D), or intergenic (I) regions of the genome. U and M represent unmethylated patterns and methylated patterns respectively. (b) Comparison of sequence features (GC content and CpG O/E) between unmethylated and methylated regions from the different genomic regions. Whole genome means sequence features of whole genome-scale DNA methylation patterns were compared. (c) Hierarchical clustering of DNA methylation patterns from different genomic regions and cell types based on histone modification signals.

A large number of reliable unmethylated regions and methylated regions at the genome scale were identified in H1, NPC, and IMR90 using CpG_MPs^37^ (Supplementary Figure S1 and Table S1). To investigate the distributions of DNA methylation patterns in different genomic regions, the whole genome-wide DNA methylation patterns were grouped into four categories according to their location: promoter, gene body, downstream, and intergenic regions (see Methods). The proportions of methylated regions in the four genomic categories were almost similar and the distributions of methylated regions were relatively stable among the three cell types (Figure 2a). However, the distributions of unmethylated regions were distinctive; in particular, the proportion of unmethylated regions in the promoter regions of IMR90 was much lower than in the other two cell types (H1: 35%, NPC: 27%, IMR90: 6%) and higher in the intergenic regions of IMR90 compared with H1 and NPC (H1: 36%, NPC: 39%, IMR90: 63%). In fact, the number of unmethylated regions in the promoter regions was similar across the different cell types (H1: 11,765, NPC: 12,210, IMR90: 13,009), whereas in the intergenic regions, the number of unmethylated regions was 8-fold higher in IMR90 compared with that in H1 and NPC. This result indicates that DNA methylation patterns in the promoter regions were relatively conserved and that new unmethylated regions occurred mostly in the intergenic regions of IMR90. Moreover, we found that about 35% of the enhancers^38^ in the intergenic regions of IMR90 occurred in the new unmethylated regions, compared to global enrichment of enhancers in human intergenic regions (p < 0.001, hypergeometric test), suggesting that the demethylation of genomic regions could be allowed to access enhancers in the distal regulatory regions of genes.

Many studies have shown that unmethylated regions tend to be enriched in high CpG density regions while methylated regions tend to be enriched in low CpG density regions^15^,^16^. In this study, however, distinct correlations were observed between sequence features (GC content and CpG observed to expected ratio (O/E)) and DNA methylation in different cell types. We found that the distributions of the sequence features between unmethylated and methylated regions were always similar and consistent with the known consensus in H1 and NPC regardless of the genomic category, but inverse trends were observed in the non-promoter regions of IMR90 maybe because of the large amounts of hypomethylated regions at the genome scale in IMR90 (Figure 2b). It suggests that the relationship between DNA methylation and genomic context is dependent both on cell type and genomic region.

The histone modifications of two DNA methylation patterns were also investigated. Sixteen histone modification signals from unmethylated and methylated patterns in the different genomic regions of each cell line were clustered using the unsupervised hierarchical clustering approach. As shown in Figure 2c, the active histone modifications and repressive modifications were grouped into two different clusters, while the same DNA methylation patterns among different cell types tended to group together. Notably, the unmethylated regions of the gene body, downstream, and intergenic regions of IMR90 were mistakenly grouped into the cluster that contained the methylated regions, indicating the correlation between DNA methylation and histone modifications may also be specific in these genome regions of IMR90.

### Prediction of DNA methylation patterns through histone modifications and sequence contexts dependent on genomic regions and cell types

The different DNA methylation patterns displayed complex sequence features and histone modification signals among the different genomic regions across the three cell types. To further investigate their correlations, we compared the performances of five common machine learning algorithms (Random forests (RF), radial basis function support vector machine (RBF-SVM), decision tree J48, naive Bayes, and logistic regression) in predicting DNA methylation patterns with the combination of sequence features and histone modifications. We found that the RF algorithm performed better than the other algorithms in relating histone modifications and sequence features with DNA methylation patterns (see Methods). Therefore, this algorithm was used to model the sequence features and/or histone modification effects on DNA methylation patterns.

**Figure 3:**
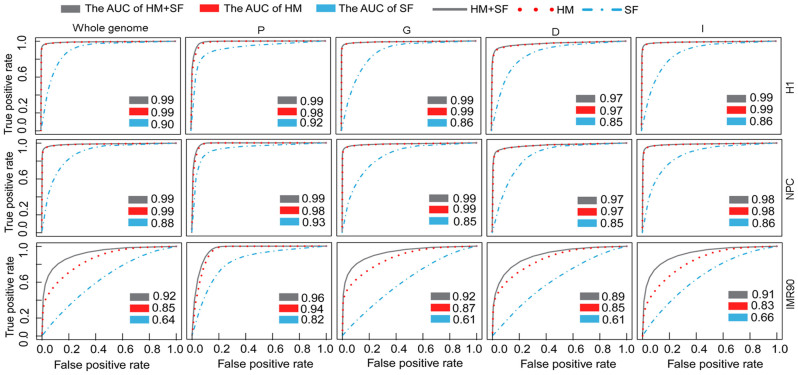
Random forests was applied to predict DNA methylation patterns. The prediction power of sequence features (SF), histone modifications (HM), and the combination of histone modifications and sequence features (HM + SF) for DNA methylation patterns by Random forests was estimated. The receiver operating characteristic (ROC) curves and the AUC for the three feature sets based on the results of ten-fold cross validation are shown. Average AUC values were used to estimate the prediction accuracy of the features.

At the whole-genome scale, the sequence features accurately modelled the DNA methylation patterns in H1 and NPC with the area under receiver operating characteristic curve (AUC) >0.88, but the accuracy was lower in IMR90 (AUC = 0.64), indicating that the prediction power of the sequence features differed among different cell types (Figure 3). The histone modifications were consistently better at predicting of DNA methylation patterns than the sequence features in the three cell types; the predictive accuracies in H1, NPC, and IMR90 were AUC = 0.99, 0.99, and 0.85 respectively. With the exception of IMR90, the predictive accuracy of the combination of histone modifications and sequence features was similar to that of histone modifications alone and was higher than sequence features alone (Figure 3). In IMR90, the combination of histone modifications and sequence features improved the accuracy by 7% (28%) compared with histone modifications (or sequence features) alone. The whole genome-scale results suggest that sequence features may be redundant when histone modifications are used to predict DNA methylation patterns in H1 and NPC, whereas they are complementary in IMR90.

Next, we compared the prediction accuracy of DNA methylation patterns in the four genomic regions. In promoters, both sequence features and histone modifications accurately predicted DNA methylation in all three cell lines (Figure 3). Moreover, the combination of sequence features and histone modifications had almost same prediction accuracy as using histone modifications alone, but the combination slightly improved the prediction accuracy compared with sequence features alone. These findings indicate that histone modifications were more closely linked with DNA methylation than with sequence features and their performances were relatively stable in different cell types. However, in the gene body, downstream, and intergenic regions, the sequence features got similar prediction accuracies in H1 and NPC (AUC >0.85) but lower accuracies in IMR90 (AUC <0.67), demonstrating that the predictive performance of sequence features was dependent on specific genomic regions and cell types. The combination of sequence features and histone modifications had similar predictive powers as histone modifications alone in H1 and NPC, and much higher predictive powers in IMR90. Thus, in H1 and NPC cells, histone modifications could discriminate unmethylated patterns and methylated patterns accurately, and little improvement was gained by including sequence features. These results imply that sequence features are relatively redundant in H1 and NPC regardless of the genomic regions. In contrast, in IMR90, the combination of sequence features and histone modifications improved the predictive accuracy depending on the specific genomic regions, indicating that the sequence features and histone modifications were complementary.

To determine the performance of the RF algorithm in predicting DNA methylation patterns based on histone modifications and sequence features, we applied our analysis method to two other DNA methylation datasets from human embryonic stem cells (H9) and peripheral blood mononuclear cells (PBMC)^10^,^39^. Sixteen and five histone modifications in H9 and PBMC, respectively, were from Encyclopedia of DNA Elements (ENCODE) by UCSD (University of California, Santa Cruz) and were used in our analysis (Supplementary Table S2). The RF algorithm performed better than the other classifying models tested (Supplementary Figure S2). Moreover, we showed that DNA methylation was more strongly correlated with histone modifications than with sequence features, in agreement with findings reported previously^9^. The accuracy of the prediction with histone modifications was more than 0.9, and little was gained by including sequence features (Supplementary Figure S3). These results further confirmed that histone modifications were indicative of genomic DNA methylation in different cell types.

### Essential histone modifications and sequence features for DNA methylation patterns with respect to different genomic regions and cell types

**Figure 4:**
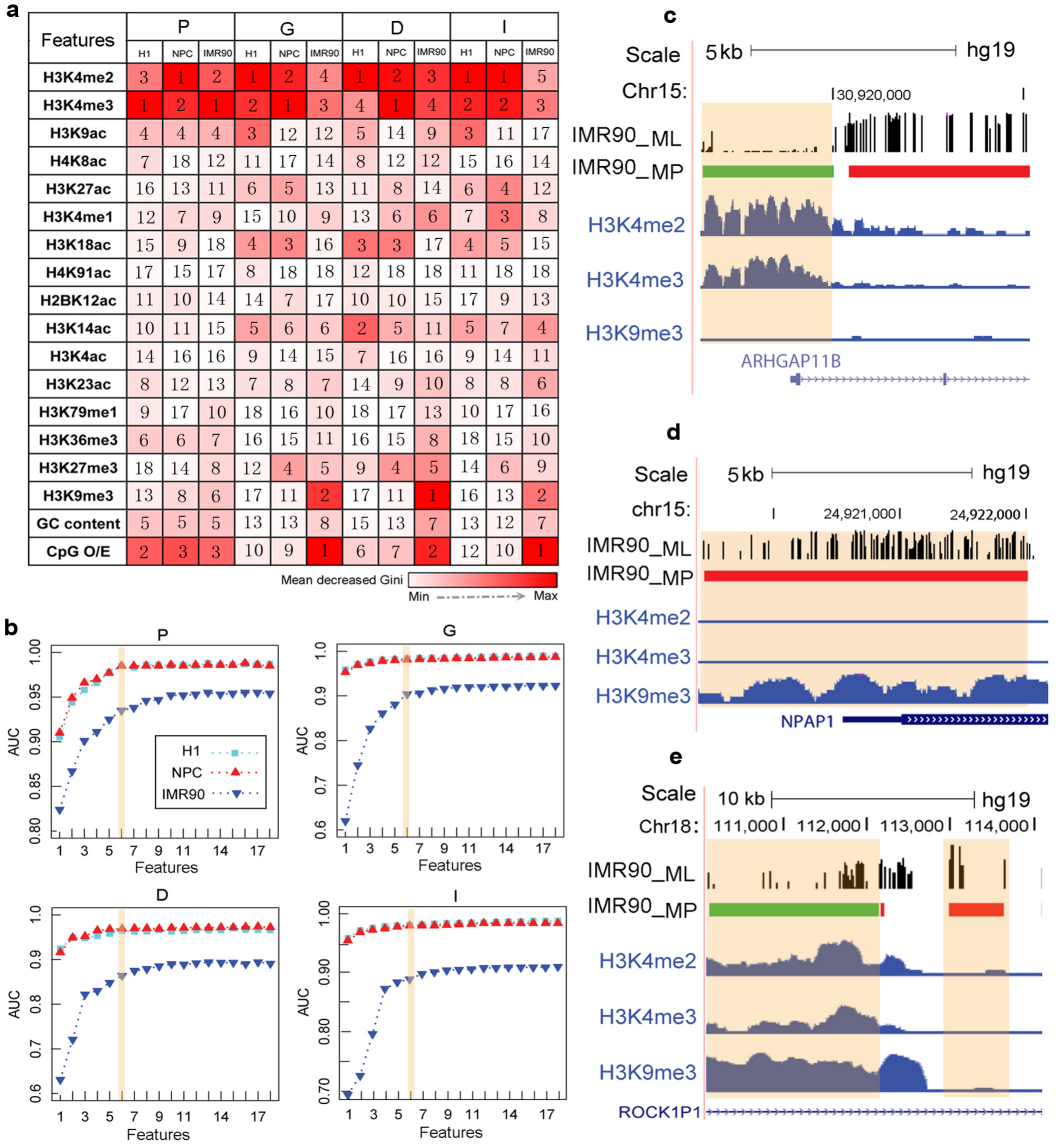
Methylation patterns of different genomic regions were predicted by various groups of features. (a) Relative importance of each feature (histone modifications and sequence features) for modelling DNA methylation patterns was quantified by mean decrease Gini (MDG) in the four genomic regions of H1, NPC, and IMR90. The feature with the largest MDG is the most important and is shown as the reddest. The number in the box corresponds to the rank of the feature. (b) Prediction accuracy of the combinations of the top *n* (*n* = 1, 2, …, 18) important features was calculated by Random forests. Eighteen features were ordered from the most important to least important, and the top six important feature combinations performed with high accuracy. The prediction accuracy was evaluated based on average AUC values. (c–e) Example showing the correlation of DNA methylation and H3K9me3 is genomic region-specific. Genomic regions located in the *ARHGAP11B* gene promoter (c) and *NPAP1* gene promoter (d) indicated that H3K9me3 and DNA methylation was positively correlated in the promoter regions. Genomic regions located in the *ROCK1P1* gene body (e) indicated that H3K9me3 was negatively correlated with DNA methylation.

To derive the key modification factors or sequence features associated with DNA methylation patterns, their relative importance for DNA methylation prediction was further examined (see Methods). Here, the mean decrease Gini (MDG) was used as the importance score of each feature to provide a relative ranking of the investigated features. The larger MDG indicates the increasing importance of the corresponding feature for the prediction of DNA methylation patterns. In the promoter regions, the top five most important features for predicting DNA methylation patterns were conserved across the three cell types. These five features were three histone modifications, H3K4me2, H3K4me3, and H3K9ac, and two sequence features, CpG O/E and GC content (Figure 4a). However, in the other three genomic regions, the key features for the prediction of DNA methylation patterns were different among the different cell types. The top two important features in H1 and NPC were H3K4me2 and H3K4me3, whereas in IMR90 they were CpG O/E and H3K9me3 (Figure 4a).

Next, we investigated the prediction powers of the combinations of different features (histone modifications and sequence features) to identify feature redundancy. Because it is computationally expensive to compare the all possible combinations, cumulative combinations of the features were employed according to a prioritized order of features for predicting DNA methylation patterns. Eighteen cumulative combinations of features were used as inputs to predict DNA methylation patterns by 10-fold cross validation using the RF algorithm. As shown in Figure 4b, the performance of the six most important features was comparable to that of the all features model (18 features) in the different genomic regions. It suggests that only a few combinations of the most important features may classify DNA methylation patterns precisely and reduce feature redundancy. The combinations of the most important six features were defined as essential factors to predict DNA methylation patterns (Supplementary Figure S4). In the promoter regions, the essential features were almost the same across the three cell types, which further illustrated the relationships between DNA methylation and histone modifications or sequence features were relatively conserved in this region. In addition, we found that sequence features were complementally grouped with histone modifications to predict DNA methylation patterns only in IMR90. H3K4me2 and H3K4me3 were the common essential factors in the different cell types and in all genomic regions, and displayed strong negative correlations with DNA methylation (Supplementary Figure S5a, Figure S5b, and Figure S6). H3K9me3 was the essential feature only in IMR90, suggesting that it may be a cell type-specific feature and that its correlation with DNA methylation may be more complex. In the promoter regions of IMR90, H3K9me3 was positively correlated with DNA methylation and inversely associated with DNA methylation in the other three genomic regions (Figure 4c, 4d, and 4e, and Supplementary Figure S5c), implying that the relationship between H3K9me3 and DNA methylation was dependent on the genomic regions.

### Modelling DNA methylation dynamics by essential histone modification changes between paired cell lines

Differentially methylated regions (DMRs) are vital clues for understanding gene transcription regulation in mammal development processes and diseases^11^,^40^. DMRs between paired cell types can be described as two patterns, from unmethylated to methylated (U→M) and from methylated to unmethylated (M→U). Differences in DNA methylation patterns cannot be fully explained by sequence features because the same sequence contexts are shared in all human cell types. In our analysis described above, we have shown that the strength of histone modification enrichment was closely related with the DNA methylation patterns. Next, we explored the possibility of predicting DMR patterns between paired cell lines based on the fold changes of the identified essential histone modifications between paired cell types.

**Figure 5:**
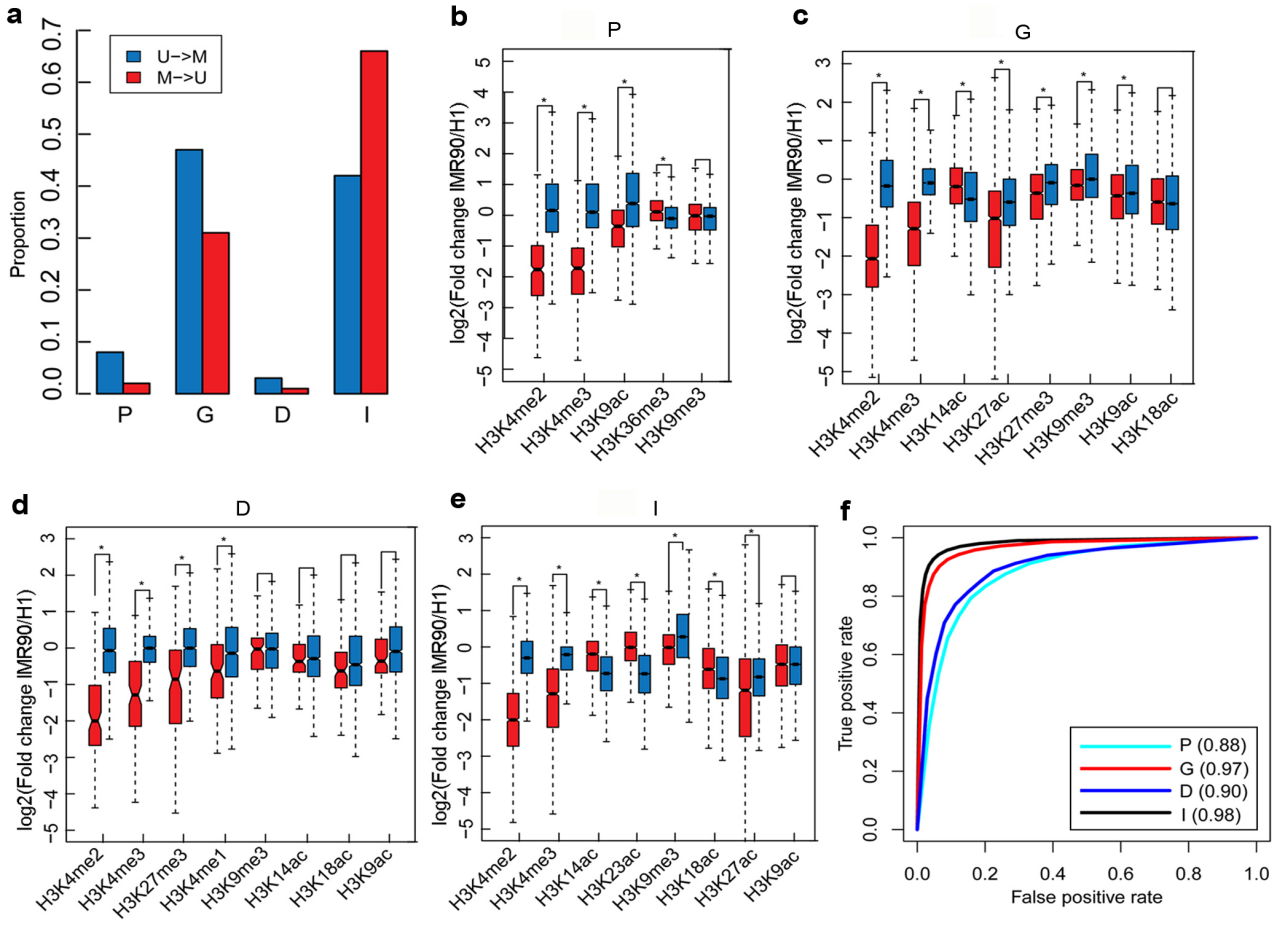
Random forests to predict DMR patterns. (a) Proportions of U→M and M→U patterns in the promoter, gene body, downstream, and intergenic regions. (b–e) Distribution of the union of essential histone modifications in H1 and IMR90 among the four genomic regions. Histone modifications that significantly differ between U→M and M→U patterns were informative effectors and used to further predict DMR patterns (p < 0.001, Wilcoxon rank sum test). (f) Prediction accuracy of DMR patterns was evaluated by 10-fold cross validation. ROC curves were plotted based on the prediction results in the promoter, gene body, downstream, and intergenic regions. The values in parentheses are the average AUC scores.

As an example, we identified DMRs by comparing H1 and IMR90 using CpG_MPs^37^. We detected 4,651 DMRs that were more highly methylated in IMR90 comparing with in H1 (U→M), and 197,993 DMRs that were less methylated in IMR90 comparing with in H1 (M→U). It is consistent with previous reports that most of the IMR90 genomic regions displayed low levels of DNA methylation^41^. We also tested whether the identified DMRs overlapped with the promoter, gene body, downstream, and intergenic regions (See Methods). Interestingly, a large fraction (58%) of U→M DMRs and 33% of M→U DMRs were associated with three gene regions (promoter, gene body, and downstream) (Figure 5a, p < 2.2e-16, chi-square test).

The essential histone modifications for predicting different DNA methylation patterns were identified across different genomic regions and cell types (Supplementary Figure S4). We then used the essential histone modifications of H1 and IMR90 to analyze the DMR patterns (Supplementary Table S3) and calculated the differences of histone modifications of DMRs between H1 and IMR90 based on the fold change [log2(IMR90/H1)]. As shown in Figure 5b–e, the variations of H3K9me3 in the M→U DMRs in the gene body and intergenic regions were stronger than the variations in the U→M DMRs in IMR90 (p < 0.001, Wilcoxon rank sum test), whereas the variations of H3K9me3 between the two DMR patterns were not statistically significant in the promoter and downstream regions (p > 0.001, Wilcoxon rank sum test). It indicates that H3K9me3 was not always correlated with DNA methylation differences among the different genomic regions. In contrast, the variations of H3K4me2 and H3K4me3 were significantly different between the two DMR patterns in the different genomic regions. Among the selected histone modifications, most of them were informative for distinguishing the DMR patterns.

The above analysis showed that differences in DNA methylation between H1 and IMR90 were related closely to changes of the essential histone modifications. Therefore, the identified informative histone modifications were applied to predict DMR patterns using the RF algorithm. Because the number of M→U DMRs (197,993) increased more than 42-fold compared with the number of U→M DMRs (4,651), we randomly sampled 4651 of the demethylated regions for the 10-fold cross validation. Here, U→M patterns were used as the positive dataset, and M→U patterns were used as the negative dataset. As a result, we found that the differences of histone modifications could precisely predict DMR patterns across different genomic regions (AUC >0.88) (Figure 5f). Thus, the variations of essential histone modifications may predict the DMR patterns between H1 and IMR90.

### Functional annotation of co-variations of epigenetic marks in different gene regions

**Figure 6:**
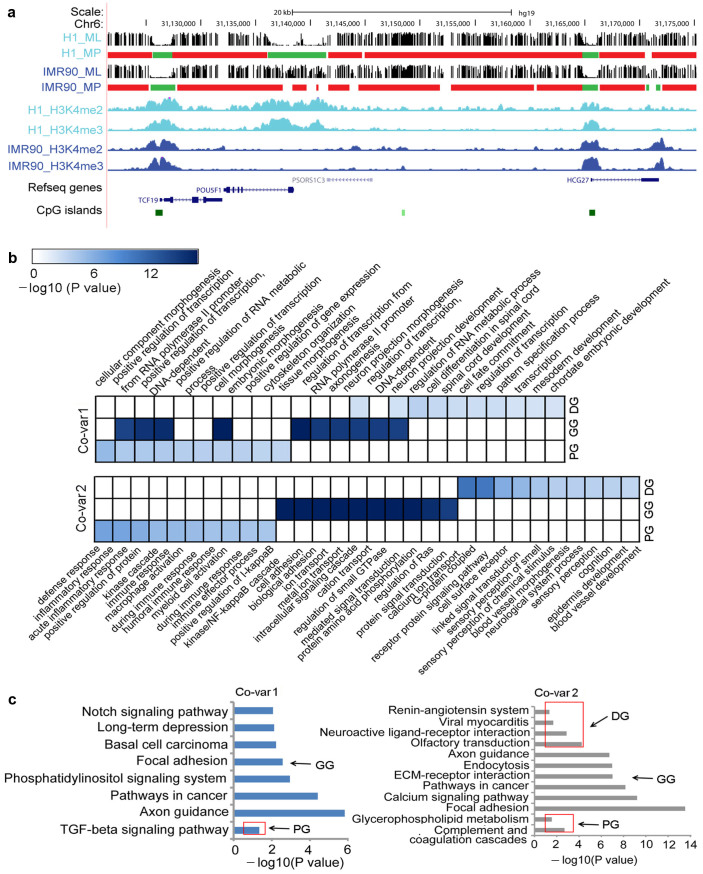
Co-variations of DNA methylation, H3K4me2, and H3K4me3. (a) Example genomic region where the variation of H3K4me2 and H3K4me3 was closely associated with the DNA methylation dynamic between H1 and IMR90. ML and MP correspond to methylation level and methylation pattern respectively. In methylation pattern, green squares represent unmethylated regions and red squares represent methylated regions. (b–c) Genes for which the co-variations of DNA methylation, H3K4me2, and H3K4me3 occurred in the promoter, gene body, and downstream regions were PG, GG and DG respectively, and were used in the enrichment analysis. Functional enrichment (p < 0.05) of GO biological process terms and KEGG pathway for three gene sets of Co-var1 and Co-var2 are showed in b and c respectively. We chose the top 10 enriched GO terms (b) and the top six enriched KEGG pathways (c) in the PG, GG, and DG regions of Covar1 and Covar2; -log10(p) was used to generate the heat map.

Among the chromatin modifications, H3K4me2 and H3K4me3 were found to be the most conserved and most important indicators of DNA methylation patterns in different genomic regions and in the different cell types. The variations of H3K4me2 and H3K4me3 signals were strongly correlated with the DNA methylation dynamics. For instance, in the promoter of the pluripotency gene *Oct4*, unmethylated regions in H1 became methylated regions in IMR90 following the decreases of H3K4me2 and H3K4me3 signals from H1 to IMR90 (Figure 6a), suggesting that these patterns could be involved in the pluripotency of cells.

Finally, to explore the functions of the different co-variations of H3K4me2, H3K4me3 and DNA methylation in the different gene regions (promoter, gene body, and downstream), gene ontology (GO) biological process terms and KEGG pathways were assigned to the genes by enrichment analyses (See Methods). Here, the co-variation with the decrease of H3K4me2 and H3K4me3 and the U→M DMRs from H1 to IMR90 was defined as Co-var1 and the opposite was defined as Co-var2 (See Methods). Co-var1s in the promoter regions were enriched in GO terms linked to cell/tissue morphogenesis and positive regulation of transcription, while neuron and spinal cord development terms were enriched in the gene body and downstream regions respectively (Figure 6b). The top GO terms for Co-var2s in the promoter regions were related to immune response, while cell adhesion and sensory of smell related functions were enriched in the gene body and downstream regions respectively. This result suggests that Co-var1 was associated mainly with development, differentiation, and gene transcription biological processes, whereas Co-var2 was related to basic biological processes. The KEGG pathway enrichment analysis showed that Co-var1s in both the promoter and gene body regions were enriched with cancer-related pathways such as TGF-beta signaling pathway and Notch signaling pathway. Co-var2s from the gene body regions were also enriched in cancer-related pathways (Figure 6c). Thus, the co-variations of epigenetic marks may provide important clues for understanding the mechanism of tumourigenesis.

### Histone modifications are conserved for DNA methylation patterns in CpG islands

**Figure 7:**
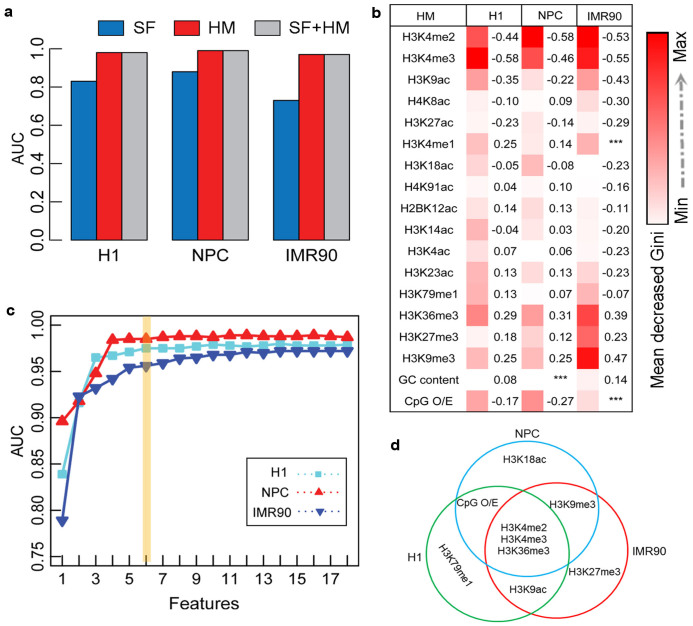
Modelling DNA methylation using histone modifications and sequence features in CpG islands. (a) Prediction accuracies of DNA methylation patterns using sequence features, histone modifications, and the combination of sequence features and histone modifications were indicated by AUC scores. (b) Heat map showing the relative importance of each feature for DNA methylation prediction. The numbers on the right are the Pearson correlation coefficients that indicate the correlation extent of DNA methylation and each of the features. (c) Prediction accuracies of the combinations of the top *n* (*n* = 1, 2, …, 18) important features. (d) Combinations of essential features for H1, NPC, and IMR90.

CpG islands are regarded as vital and conserved unmethylated regions across various normal cell types, and their abnormal DNA methylation has been linked closely with disease occurrence^42^,^43^. Recent studies using BisChIP-seq have shown a distinct relationship between DNA methylation and H3K27me3 in CpG islands^33^,^34^. To investigate their interactions in CpG islands, we applied the RF algorithm to predict DNA methylation patterns with the corresponding 16 histone modifications and sequence features. We obtained a high prediction accuracy of histone modifications (AUC >0.96) in the CpG islands in all three cell types (Figure 7a). The prediction accuracy of the sequence features was lower; AUCs = 0.83, 0.88, and 0.73 in H1, NPC, and IMR90 respectively. Histone modifications accurately predicted the CpG islands DNA methylation patterns and the combination of histone modifications and sequence features produced similar results (Figure 7a), which is similar in the promoter regions. It suggests that the relationship between DNA methylation patterns and histone modifications may be much closer and more conserved than the relationship with sequence features in the promoter regions and CpG islands.

The relative importance of chromatin modifications and sequence features for DNA methylation prediction in CpG islands shows that H3K4me2 or H3K4me3 were still the most important features (Figure 7b). It's consistent with previous studies that showed H3K4 methylation was associated with the maintenance of the CpG islands unmethylated patterns^14^. Repressive H3K9me3 mark was stably positively correlated with DNA methylation in the promoter regions and in CpG islands (Figure 7b and Supplementary Figure S5c). Inactive CpG islands promoter regions are thought to be regulated by DNA methylation, a long-term repressive state^39^,^41^. Methylated promoters are occupied by nucleosomes at TSSs that are enriched with H3K9me3 and stabilized by methylated DNA-binding proteins, which in turn recruit histone deacetylases to the region^44^. We also extracted the essential features for DNA methylation prediction of CpG islands. Figure 7c shows that only the most essential six feature combinations could correctly classify the CpG islands DNA methylation patterns in H1, NPC, and IMR90. Among these combinations, H3K4m2, H3K4me3, and H3K36me3 were common essential factors in the three cell types, suggesting that H3K36me3 may also be conserved in CpG islands (Figure 7d). Moreover, the essential factors in the CpG islands were also predictive of the DMR patterns between H1 and IMR90 (AUC = 0.91) (Supplementary Figure S7).

## Discussion

In this work, we investigated the relative relationship of histone modifications and genomic sequence contexts to DNA methylation patterns in H1, NPC, and IMR90 based on the RF classifier. Although previous studies have found that both histone modifications and sequence features were correlated with DNA methylation, our work provides a genome-wide insight into their genomic region-specific and cell type-specific relationships.

Recently, many whole genome DNA methylation profiles have been produced by BS-seq, and the ENCODE project has provided a wealth of histone modification profiles by ChIP-Seq. The wide range of data allowed us to employ computational methods on a whole genome-scale and in different functional genomic regions, without being limited to only CpG islands or promoters^9^,^45^.

The association between DNA methylation and histone modifications or sequence features was similar in H1 and NPC, but, in IMR90, the association was specific to the gene body, downstream, or intergenic regions. We found that additional information was gained by including sequence features only in these three regions by comparing the performance of histone modifications with the performance of the combination of histone modifications and sequence features. Thus, the epigenetic state seemed to have experienced important remodelling during development, and the mechanisms involved in regulating the association of histone modifications and DNA methylation may be different in IMR90. The active histone modification signals of unmethylated patterns in IMR90 decreased markedly compared with in the other three cell lines (p < 0.05, Wilcoxon rank sum test). In contrast, repressive histone modifications H3K27me3 and H3K9me3 signals were much stronger and may be associated with the loss of pluripotency in IMR90^18^,^30^. Compared with differentiated cell types, the epigenetic status is an open chromatin structure with characteristic methylation and histone modification maps in embryonic stem cells^30^. H3K9me3 domains in these cells are small and interspersed, but are substantially expanded in IMR90^18^, implying that the relationship between DNA methylation and histone modifications is nuanced and complex during development. Illustrating their associations may help improve the understanding of somatic cell reprogramming.

We employed the RF algorithm to further confirm that histone modifications were predictive of the dynamics of DNA methylation between paired cell types, and also compared H1 to NPC. Compared with the relationship between H1 and IMR90, NPC is derived directly from H1; therefore, the DNA methylation landscapes of these two cell types do not change dramatically, meaning that the DMRs are relative less. For example, the number of U→M patterns in the promoter and downstream regions was only 72 and 27 respectively (Supplementary Table S4). The datasets of the promoter and downstream regions were small, making it difficult to obtain stable and reliable prediction accuracies by ten-fold cross validation using RF. Therefore, to predict whole genome DMRs between H1 and NPC, we used the variations of the 16 histone modifications and obtained a highly accuracy (AUC = 0.93) (Supplementary Figure S8). We have shown that the variations of histone modifications may also be used to predict the DNA methylation dynamics between H1 and NPC. These results suggest that the co-variations of epigenetic marks are important clues for cellular identity. By applying this method to find the co-variation relevant genes between normal and cancer cell types may help to obtain potential cancer-related marks. Therefore, it is essential to identify the co-variations of paired cell types to gain new understanding of biological processes from the large amounts of data that is now publicly available.

## Methods

### DNA methylation and histone modification datasets

We collected genome-wide DNA methylation and 16 histone modification profiles in three cell types: embryonic stem cells (H1), H1-derived neuronal progenitor cultured cells (NPC), and foetal fibroblasts (IMR90). The DNA methylation profiles were measured by whole-genome shotgun BS-seq, which provides comprehensive single-nucleotide resolution DNA methylation. DNA methylation maps of H1 and IMR90 were downloaded from http://neomorph.salk.edu/human_methylome/41, and a DNA methylation map of NPC was obtained from the ENCODE project by UCSD^46^. Bismark^47^ was used to map the BS-seq reads onto the human reference genome (hg19) and the corresponding methylation information was obtained. The common 16 histone modifications of H1, NPC, and IMR90 were sourced from ENCODE (H3K4me1, H3K4me2, H3K4me3, H3K79me1, H3K27me3, H3K9me3, H3K36me3, H3K9ac, H3K27ac, H2BK12ac, H3K4ac, H4K8ac, H4K91ac, H3K23ac, H3K14ac, and H3K18ac)^46^. Histone modification profiles were detected by ChIP-seq and their sequencing reads were aligned to the hg19 sequence.

### Identify genome-wide methylation patterns and classify them into different genomic regions

CpG_MPs was developed to accurately identify genome-wide unmethylated regions and methylated regions from high-throughput BS-seq methylation profiles based on a hotspot extension algorithm^37^. CpG_MPs was applied to identify the DNA methylation patterns of H1, NPC, and IMR90.

To evaluate whether the correlation between DNA methylation and features of interest (histone modifications and sequence features) was consistent across different genomic regions, we classified the DNA methylation patterns into four genome-wide categories: promoter, gene body, downstream, and intergenic regions. Promoter regions were centred [−1000 bp, +500 bp] to a TSS; gene body regions were centred in the region >[+500 bp] to a TSS and <[−500 bp] to the corresponding TTS; downstream regions were centred [−500 bp, +1000 bp] to the corresponding TTS; and intergenic regions were the regions that remained after the other three regions were classified.

### Sequence features and histone modification signals of DNA methylation patterns

The human reference genome (hg19) was downloaded from the UCSC and the sequence features including GC content and CpG O/E (observed/expected) were calculated by CpG_MPs^37^. The GC content and CpG O/E of a genomic region were determined as:
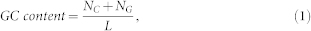

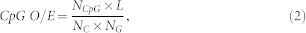
where *L* is the length of the genomic region, and *N_C_*, *N_G_*, and *N_CpG_* are the number of cytosine, guanine, and CpG in the investigated genomic region respectively.

RPKM (reads assigned per kilobase of target per million mapped reads) is normally used to quantify gene expression level from RNA-seq^48^. Here, we calculated RPKM as histone modification signals according to the method described by Hon et al^49^. To avoid RPKM being equal to zero, a pseudocount was added to *Reads* and was calculated as:
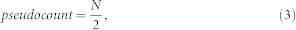
where *N* represents the total number of histone modification reads in the experiment. Each histone modification signal in a genomic region was defined as:
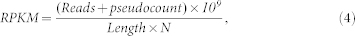
where *Reads* represents the number of corresponding histone modification reads located in the genomic region, and *Length* is the number of nucleotides in the genomic region.

### Robust model for predicting DNA methylation patterns

Five frequently-used machine learning classification algorithms (RF, RBF-SVM, decision tree J48, naive Bayes, and logistic regression) were used to predict DNA methylation patterns using WEKA^50^ with the sequence features and histone modifications. Here, we compared the performances of the five algorithms using the methylated regions as the positive dataset and the unmethylated regions as the negative dataset. We randomly sampled the same number of unmethylated regions and methylated regions from whole genome-wide DNA methylation patterns to avoid bias caused by an unbalanced proportion of positive and negative datasets, and performed 10-fold cross validation for the sampled datasets to estimate the model accuracy. The process was repeated ten times and the average area under the receiver operating characteristic curve (AUC) was computed as the major indicator of prediction accuracy (Supplementary Figure S9). To further evaluate the models, we also calculated accuracy (ACC), sensitivity (SE), and specificity (SP) for each model. The RF algorithm achieved the highest accuracy (AUC >0.90) and obtained comparable accuracy, sensitivity, and specificity values across H1, NPC, and IMR90 (Table 1 and Supplementary Figure S10). Therefore, RF was used to integrate the histone modifications and sequence features for DNA methylation prediction.

We also compared the performance of sequence features (GC content and CpG O/E), histone modifications (16 histone modifications), and the combination of sequence features and histone modifications (GC content, CpG O/E and 16 histone modifications) genome-wide and in the different genomic regions. We used the RandomForest R package to evaluate the importance of features for classifying DNA methylation patterns by MDG^51^. At each node of RF, a low Gini indicates that samples are well classified. Then MDG of all the nodes in all trees in the forest was used to evaluate the overall discriminative power of a particular feature for the classification.

### Identify differentially methylated regions

CpG_MPs was applied to identify DMRs between H1 and IMR90^37^. We classified DMRs into U→M pattern (DMR in H1 is unmethylated and methylated in IMR90) and M→U pattern (DMR in H1 is methylated and unmethylated in IMR90). Sequence features and histone modification signals of DMRs were also computed. To predict DNA methylation dynamics between paired cell lines by RF, we used U→M as the positive dataset and M→U as the negative dataset. We also performed 10-fold cross validation by sampling the same number of U→M and M→U DMRs in a random manner, as described above for the unmethylated and methylated regions. The process was repeated 10 times and the average AUC, ACC, SE, and SP values were calculated to evaluate the predictive performance.

### Gene Ontology analysis

U→M patterns and M→U patterns are characterized with distinctive H3K4me2 and H3K4me3 marks. In the promoter regions, we chose U→M patterns with variations of H3K4me2 (log_2_(IMR90_H3K4me2_/H1_H3K4me2_)) and H3K4me3 (log_2_(IMR90_H3K4me3_/H1_H3K4me3_)) that were less than their three-quarters quantile and M→U patterns with variations of H3K4me2 (log_2_(IMR90_H3K4me2_/H1_H3K4me2_)) and H3K4me3 (log_2_(IMR90_H3K4me3_/H1_H3K4me3_)) that were higher than their one-quarter quantile. Genes that corresponded to the promoter U→M DMRs or M→U DMRs were obtained. Genes relevant to the gene body and downstream regions were also identified as promoters. These different gene sets were assigned GO terms and KEGG pathways by enrichment analysis using DAVID^52^.

### CpG island analysis

CpG islands are important epigenetic regulatory elements and abnormal methylation patterns in these regions are associated with cancer^53^. To analyze DNA methylation patterns in CpG islands, we downloaded the CpG islands data from UCSC http://genome.ucsc.edu/. DNA methylation patterns centred in CpG islands were grouped into CpG islands category.

## Author Contributions

Y.Z., J.S., H.Y. and Q.W. designed the study. H.Y. and J.S. performed the data analysis and drafted the manuscript. H.L., Y.W. and J.L. performed the statistical analysis. F.W., C.Z. and G.Z. interpreted the function annotations. All authors read and approved the final manuscript.

## Supplementary Material

Supplementary Information Supplementary Information

## Figures and Tables

**Table 1:** Performance of five machine learning algorithms for DNA methylation prediction

Cell line	Random forests	RBF-SVM	Decision tree J48	Naive Bayes	Logistic regression
H1	0.991	0.972	0.977	0.951	0.980
NPC	0.987	0.961	0.974	0.942	0.983
IMR90	0.920	0.854	0.891	0.695	0.826

The numbers are the average AUC scores that were used to evaluate the prediction accuracy of the algorithms.
