# Supplementary material for: Chromatin modifications and genomic contexts linked to dynamic DNA methylation patterns across human cell types
Source: Sci Rep. 2015 Feb 12;5:8410. doi: 10.1038/srep08410 (PMC4325317; doi:10.1038/srep08410)
Supplement: Supplementary Information [file srep08410-s1.pdf]

Chromatin modifications and genomic contexts linked to dynamic DNA methylation patterns across human cell types

Haidan Yan, Dongwei Zhang, Hongbo Liu, Yanjun Wei, Jie Lv, Fang Wang, Chunlong Zhang, Qiong Wu, Jianzhong Su, and Yan Zhang

Contents

1 Supplementary Figures and Tables

1 Supplementary Figures and Tables

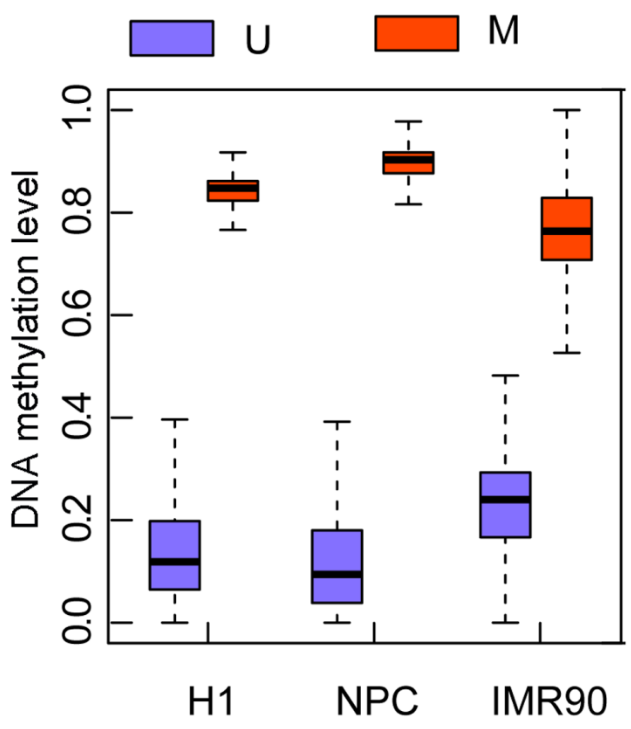

Supplementary Figure S1: Comparison of average methylation levels between unmethylated regions (URs) and methylated regions (MRs) in H1, NPC and IMR90.

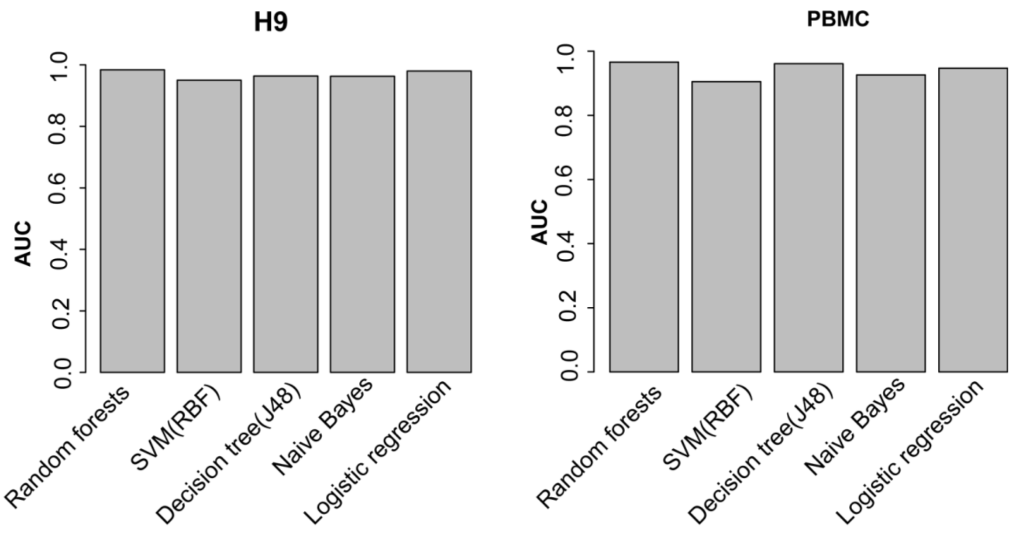

Supplementary Figure S2: The prediction accuracy of DNA methylation in H9 and PBMC based on five machine learning algorithms. ACC, SE and SP represent accuracy, sensitivity and specificity respectively.

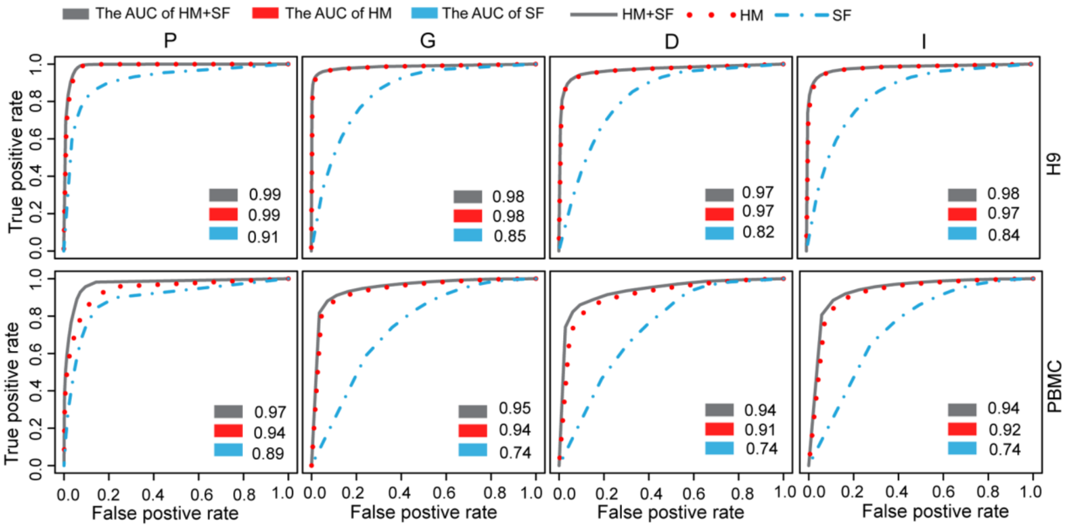

Supplementary Figure S3: Comparing the prediction accuracy of HM, SF and HM+SF in H9 and PBMC.

Essential features

P			G			D			I		
H1	NPC	IMR90	H1	NPC	IMR90	H1	NPC	IMR90	H1	NPC	IMR90
H3K4me3	H3K4me2	H3K4me3	H3K4me2	H3K4me3	CpG O/E	H3K4me2	H3K4me3	H3K9me3	H3K4me2	H3K4me2	CpG O/E
CpG O/E	H3K4me3	H3K4me2	H3K4me3	H3K4me2	H3K9me3	H3K14ac	H3K4me2	CpG O/E	H3K4me3	H3K4me3	H3K9me3
H3K4me2	CpG O/E	CpG O/E	H3K9ac	H3K18ac	H3K4me3	H3K18ac	H3K18ac	H3K4me2	H3K9ac	H3K4me1	H3K4me3
H3K9ac	H3K9ac	H3K9ac	H3K18ac	H3K27me3	H3K4me2	H3K4me3	H3K27me3	H3K4me3	H3K18ac	H3K27ac	H3K14ac
GC content	GC content	GC content	H3K14ac	H3K27ac	H3K27me3	H3K9ac	H3K14ac	H3K27me3	H3K14ac	H3K18ac	H3K4me2
H3K36me3	H3K36me3	H3K9me3	H3K27ac	H3K14ac	H3K14ac	CpG O/E	H3K4me1	H3K4me1	H3K27ac	H3K27me3	H3K23ac

Supplementary Figure S4: The identified essential features including histone modifications and sequence features for predicting DNA methylation patterns in different genomic regions and cell types.

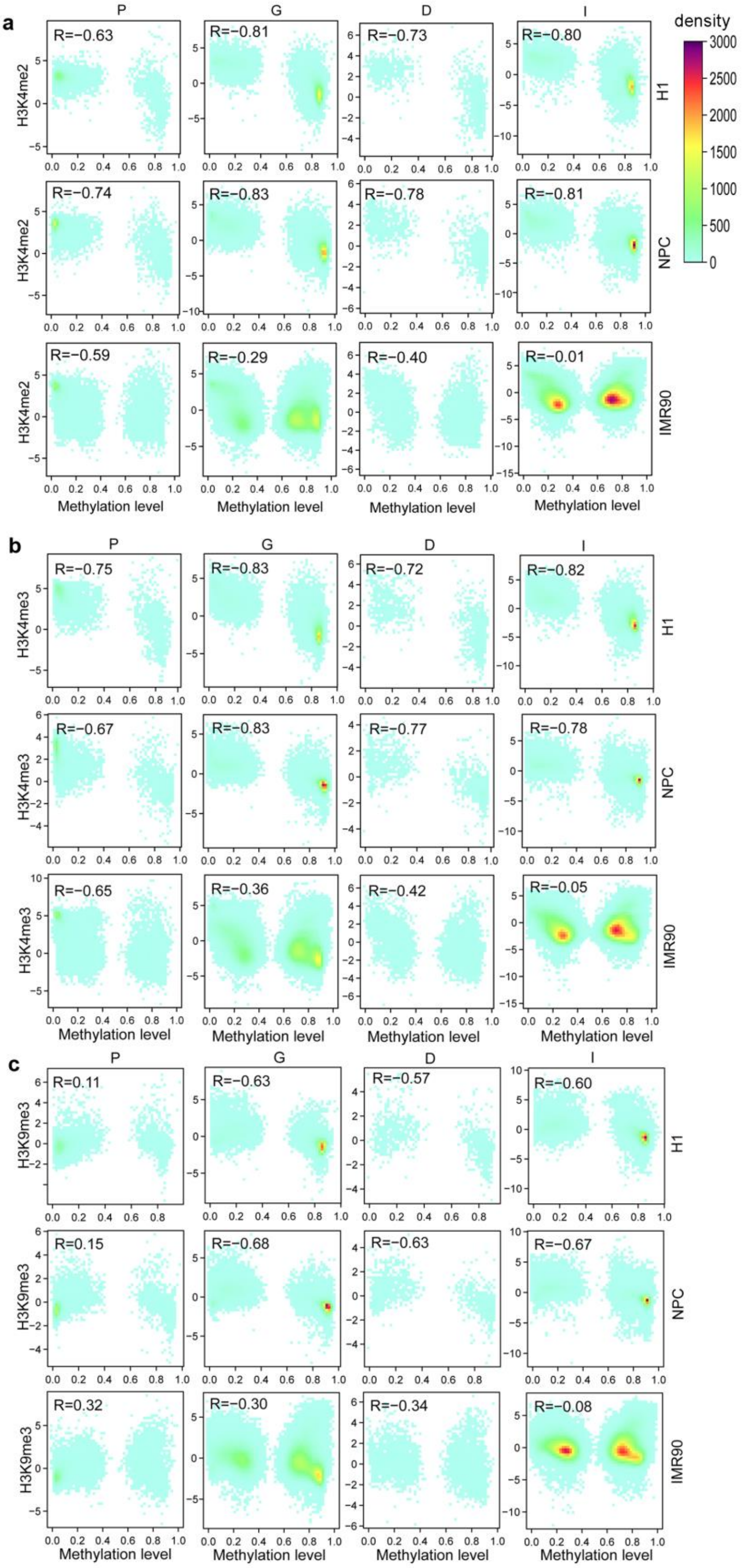

Supplementary Figure S5: The correlation between DNA methylation and H3K4me2, H3K4me3 and H3K9me3 across promoter, genebody, downstream and intergenic regions in H1, NPC and IMR90.

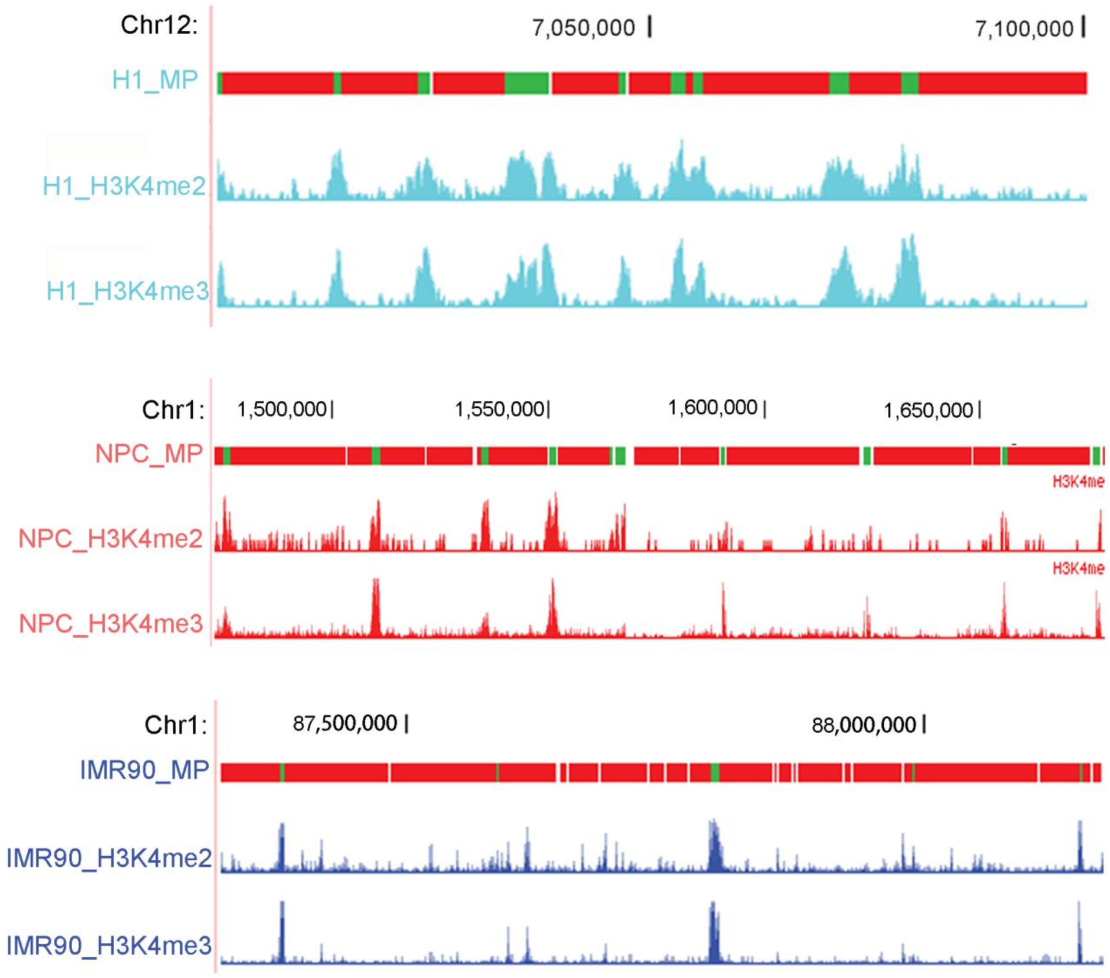

Supplementary Figure S6: Example regions showed that DNA methylation and H3K4me2/H3K4me3 is negative-correlation.

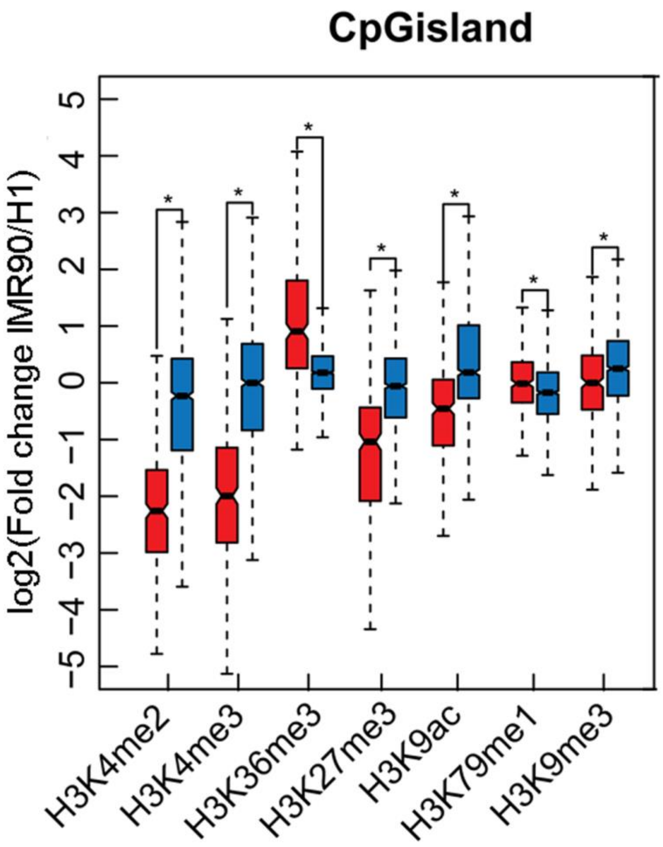

Supplementary Figure S7: the distributions of the union of essential histone modifications in H1 and IMR90 for CpG islands. The red is for Co-var1, the blue is Co-var2.

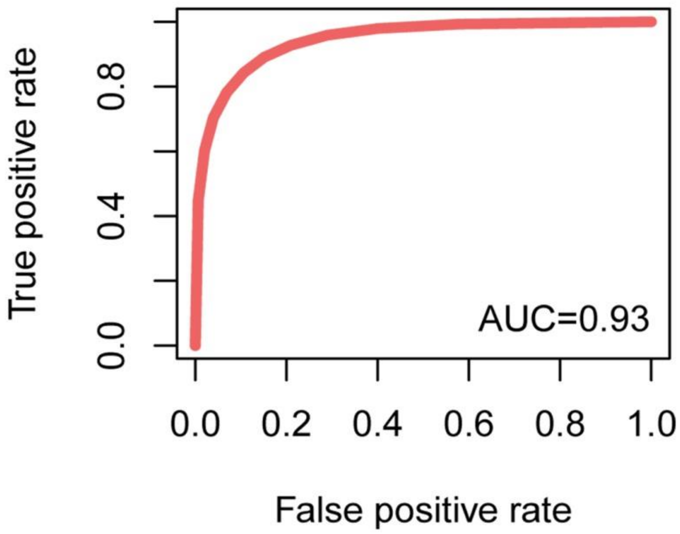

Supplementary Figure S8: The prediction AUC of DMR patterns between H1 and NPC by histone modifications using ten-fold cross validation.

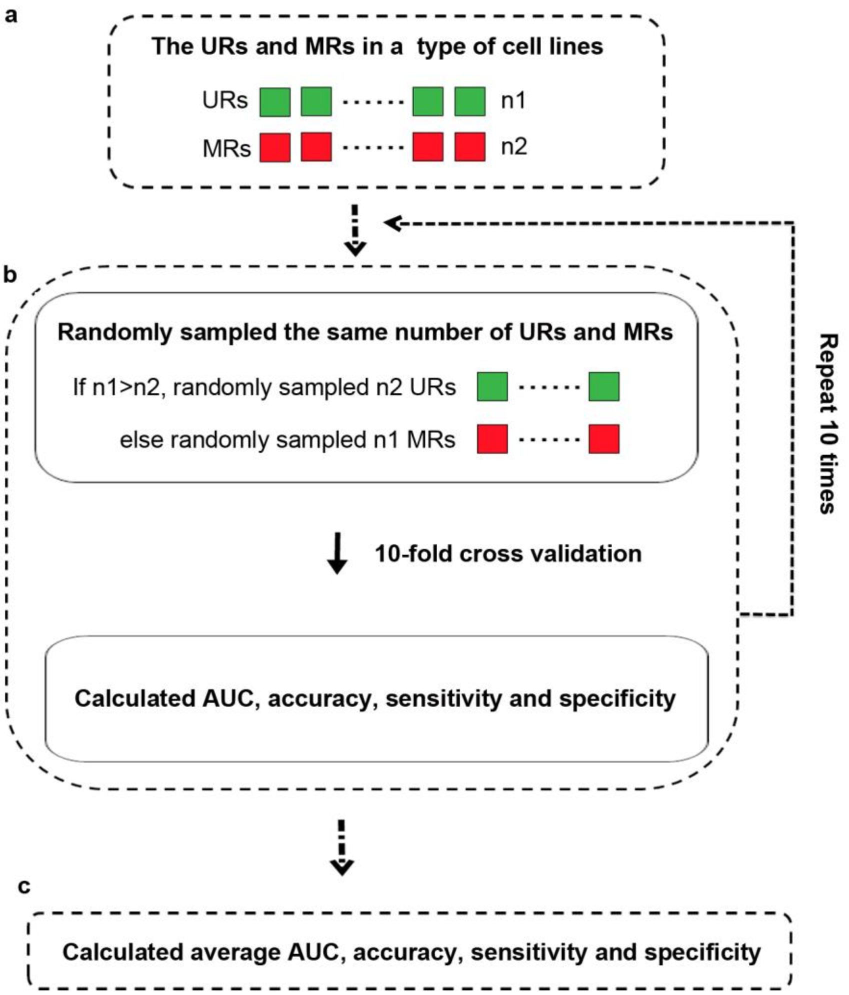

Supplementary Figure S9: The flowchart of calculated average AUC, accuracy, sensitivity and specificity by 10-fold cross validation. (a) The unmethylated regions (URs) and methylated regions (MRs) identified in a type of cell lines. n1 and n2 represent the number of URs and MRs respectively. (b) Randomly sampled the same number of URs and MRs to perform 10-fold cross validation by machine learning algorithms and calculated the AUC, accuracy, sensitivity and specificity. To get reliable model accuracy, we repeated the process 10 times. (c) The average AUC, accuracy, sensitivity and specificity were calculated to estimate the prediction accuracy.

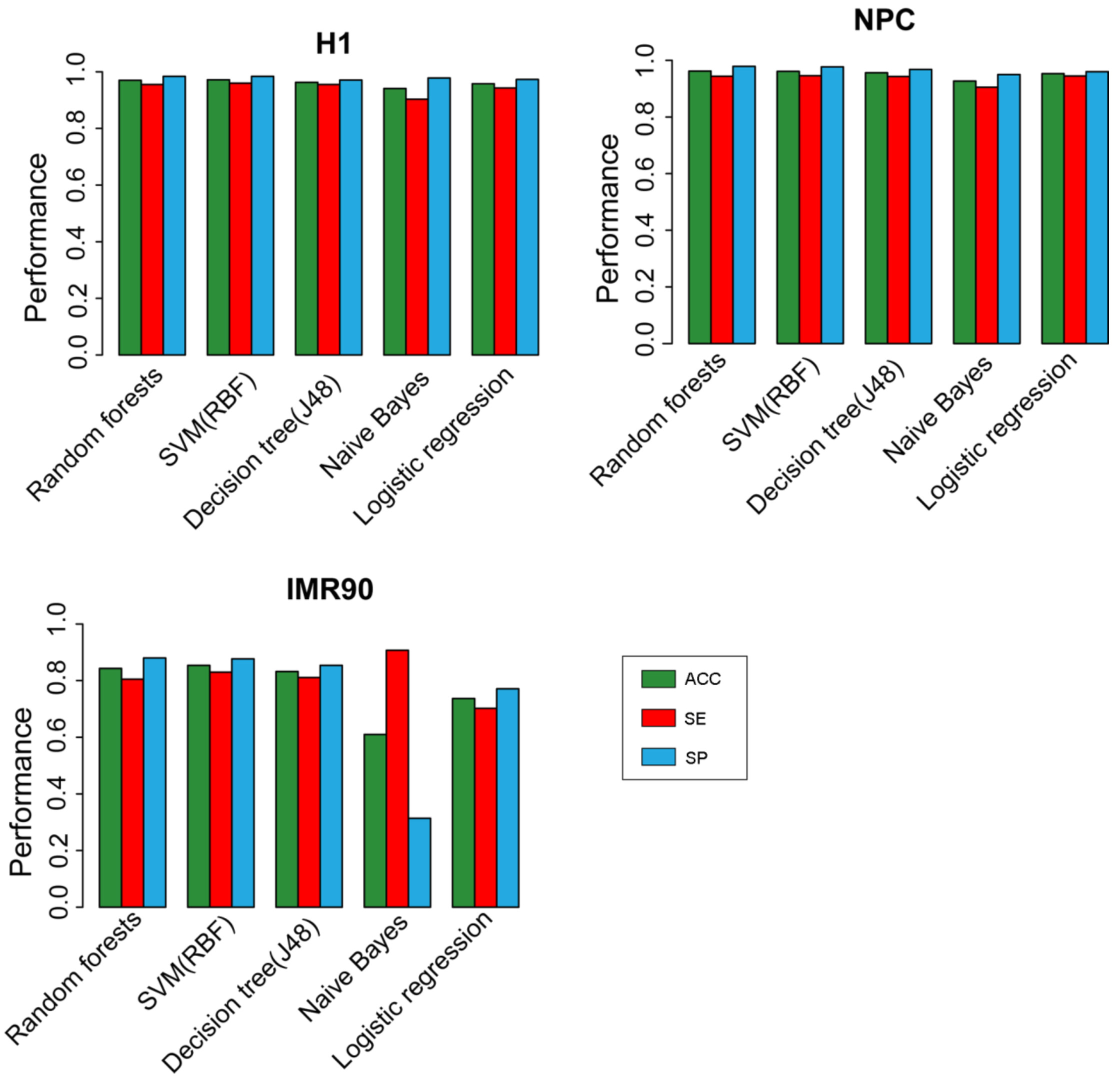

Supplementary Figure S10: The performance of different machine learning algorithms for predicting DNA methylation across H1, NPC and IMR90.

Supplementary Tables:

Supplementary Table S1: The identified unmethylated and methylated regions by CpG_MPs from H1, NPC and IMR90.

Cell line	Unmethylated regions					Methylated regions				
	Number	Length	DML*	G+C*	CpG*	Number	Length	DML*	G+C*	CpG*
H1	33326	1283	0.14	0.57	0.61	66580	43775	0.83	0.44	0.48
NPC	45556	1149	0.12	0.56	0.66	87179	32825	0.88	0.44	0.33
IMR90	233045	2470	0.23	0.44	0.30	348008	4615	0.77	0.45	0.35

DML* represents the average DNA methylation level of corresponding DNA methylation pattern. G+C* and CpG* represent G+C content and CpG O/E, respectively.

Supplementary Table S2: The histone modifications of H9 and PBMC.

Cell types	Histone modifications
H9	H3K4me1, H3K4me2, H3K4me3, H3K79me1, H3K27me3, H3K9me3, H3K36me3, H3K9ac, H3K27ac, H2BK12ac, H3K4ac, H4K8ac, H4K91ac, H3K23ac, H3K14ac, H3K18ac
PBMC	H3K27me3, H3K9me3, H3K36me3, H3K9ac, H3K4me1

Supplementary Table S3: The union of essential histone modifications of H1 and IMR90.

Genomic regions	Histone modifications
P	H3K4me2,H3K4me3,H3K9ac,H3K36me3,H3K9me3
G	H3K4me2,H3K4me3,H3K9ac,H3K18ac,H3K14ac,H3K27ac,H3K9me3,H3K27me3
D	H3K4me1,H3K4me2,H3K4me3,H3K9ac,H3K14ac,H3K18ac,H3K9me3,H3K27me3
I	H3K4me2,H3K4me3,H3K9ac,H3K14ac,H3K18ac,H3K27ac,H3K23ac,H3K9me3

Supplementary Table S4: the number of U→M and M→U patterns located in promoter (P), genebody (G), Downstream (D) and intergenic regions (I).

Genome	U→M				M→U			
	P	G	D	I	P	G	D	I
H1->NPC	72	528	27	784	233	3247	77	3063
H1->IMR90	395	2170	115	1971	3357	61106	1508	132022
